# Physiology in Africa: Reflections on the past to imagine future possibilities

**DOI:** 10.14814/phy2.70046

**Published:** 2024-09-09

**Authors:** M. Faadiel Essop

**Affiliations:** ^1^ Director of the Centre for Cardio‐Metabolic Research in Africa (CARMA), Division of Medical Physiology, Faculty of Medicine and Health Sciences Stellenbosch University Cape Town South Africa

This invited editorial provides an opportunity to reflect on the origins and current activities of the African Association of Physiological Sciences (AAPS) as well as some recent progress. Future paths are also considered to ensure the sustainability and further development of Physiology on our continent.

The AAPS was established on July 8, 1989 with the aim of training young physiologists, enhancing teaching and learning practices, and pursuing biomedical research endeavors relevant to Africa's needs (Saeed & Ebeigbe, 2013). However, initial progress was relatively stunted despite such worthy aims. Of note, the 6th AAPS congress of 2012 in Egypt was a seminal gathering as there was a renewed commitment by affiliate societies (Egypt, Nigeria, Sudan, South Africa) to cooperate in a coherent/collective manner to truly realize the 1989 ideals. As a newly elected council member I felt a sense of optimism as we launched the Journal of the AAPS (JAAPS) in 2013 with the late Prof. Anthony Ebeigbe acting as its first Editor‐in Chief.

I was subsequently elected as the AAPS president in 2021, and after strategic sessions with council, we embarked on a renewal process to ensure a thriving/dynamic society that genuinely served the needs of African physiologists. The AAPS vision was re‐evaluated and now states our intent to: *advancing world class research and promoting teaching of physiological sciences in Africa*. Moreover, we agreed on six mission indicators that strongly guide our planning, decision‐making, and actions: (a) strengthen organizational capacity, and cooperation between continental and related global societies; (b) promote research capacity and collaboration between African physiologists and others; (c) enhance teaching and learning skills development of physiologists in Africa and others; (d) encourage capacity building (of especially young physiologists) and the networking of scientists in Africa; (e) facilitate information exchanges by arranging congresses, workshops and meetings; and (f) promote communication and outreach between African physiologists and the wider scientific community and the public.

The progress made since 2021 has been remarkable, for example, the number of affiliate societies increased from the original four to 19 and includes Ethiopia, Kenya, Rwanda, Tanzania, Uganda, Zambia, francophone countries such as Benin, Gabon, Gambia, Ivory Coast, Niger, Senegal, and individual memberships in countries where no societies currently exist. There are currently 1257 physiologists listed on our newly established database that details, for example, researcher expertise, contact details, qualifications, and laboratory equipment (https://aapsnet.org/map/). Here, the idea is to readily make available such information to enhance communication/collaboration between African physiologists and others. The AAPS constitution was also revised to facilitate the election of a more representative and federal‐like council that should ensure inputs from all its affiliate societies. The new council will be elected later this year and will be formally inducted at the next AAPS congress (Ismailia, Egypt; 13–15 February 2025). In addition, the Young AAPS (YAAPS) was recently launched to empower young physiologists to become part of decision‐making and to grow our next leadership cohort. There has also been significant progress in terms of promoting teaching and learning activities, with the AAPS formally adopting the PhysioCAFUN curriculum (Alagbonsi et al., 2024) that is a freely available resource (https://aapsnet.org/documents/).

However, significant challenges remain regarding the future of Physiology in Africa. When considering research development and innovation, these include, for example, lack of adequate grant funding in several countries, limited studies identifying African‐related disease risk factors, suboptimal research facilities and equipment in several tertiary institutions, and the brain drain of African physiologists (Oyeniran et al., 2021). Moreover, an analysis of the AAPS database revealed that 64% of the listed African physiologists possess a PhD degree and that this is skewed toward relatively wealthier countries (e.g., South Africa and Nigeria) that spend greater amounts of their GDP on research and development. Thus, the relatively low number of principal investigators/lecturers with a doctoral degree means there is a limited capacity to grow postgraduate student cohorts across Africa.

In terms of focusing on African‐related health challenges, our database analyses showed that there is a strong focus on noncommunicable diseases (e.g., cardiovascular and cancers). Such research efforts are to be commended as this is well aligned with current data showing that noncommunicable diseases are overtaking infectious diseases by accounting for ~37% of the African disease burden (World Health Organization, 2019). Although communicable and parasitic diseases remain a major burden of disease, it is our opinion that the intersection of infectious diseases (such as HIV) with noncommunicable diseases should also be vigorously pursued in terms of the African research agenda (Dominick et al., 2020).

Regarding teaching and learning, the relatively low number of suitably qualified principal investigators and supervisors for postgraduate student supervision is a concern, while at undergraduate level, low lecturer‐to‐student ratios persist in many countries (Sofola, 2014). In some institutions, the lack of adequate information communications technology, poor infrastructure, and outdated curricula makes innovation and cutting‐edge approaches in teaching and learning a lot more challenging.

How can we begin to deal with such challenges? One way forward would be to establish a network of research centers of excellence to allow for collaborative projects, sharing of resources and promoting student exchanges on the African continent (El‐Wazir, 2015). Here, the ideal should be to aim for around four (existing) research centers of excellence to jointly launch a few big projects that will investigate major African health issues, including the concerning effects of climate change. Such efforts can also be coupled to state‐of‐the‐art teaching and learning of postgraduate students, and in the future be expanded to include lecturer training and development. Global North–South collaborations should be encouraged and be pursued in an equitable manner to create win‐win scenarios. Such partnerships should assist to develop a critical mass of younger physiologists with the necessary skills and training to further grow the discipline of Physiology in Africa. Of note, the IUPS recently launched a mentor–mentee scheme that will certainly benefit young African physiologists. However, governments should be more intensely lobbied by learned societies to ensure a greater proportion of budgets are spent on research and development to retain such young talent and to thereby limit the detrimental brain drain.

## FUNDING INFORMATION

No funding information provided.

## ETHICS STATEMENT

The author is the President of the African Association of Physiological Sciences (AAPS). The author is a member of the Editorial Board of Physiological Reports and was not involved in reviewing or making decisions for this manuscript.
